# 1,3-Thia­zole-4-carbo­nitrile

**DOI:** 10.1107/S2414314621013328

**Published:** 2021-12-21

**Authors:** Martin J. G. Fait, Anke Spannenberg, Evgenii V. Kondratenko, David Linke

**Affiliations:** a Leibniz-Institut für Katalyse e. V., Albert-Einstein-Str. 29a, 18059 Rostock, Germany; University of Aberdeen, Scotland

**Keywords:** crystal structure, thia­zole, nitrile group, hydrogen bonding, π–π stacking inter­action

## Abstract

The title compound, C_4_H_2_N_2_S, is a 1,3-thia­zole substituted in the 4-position by a nitrile group. In the crystal, C—H⋯N hydrogen bonds and aromatic π–π stacking inter­actions are observed.

## Abstract 2

The title compound, C_4_H_2_N_2_S, is a 1,3-thia­zole substituted in the 4-position by a nitrile group.

## Graphical abstract

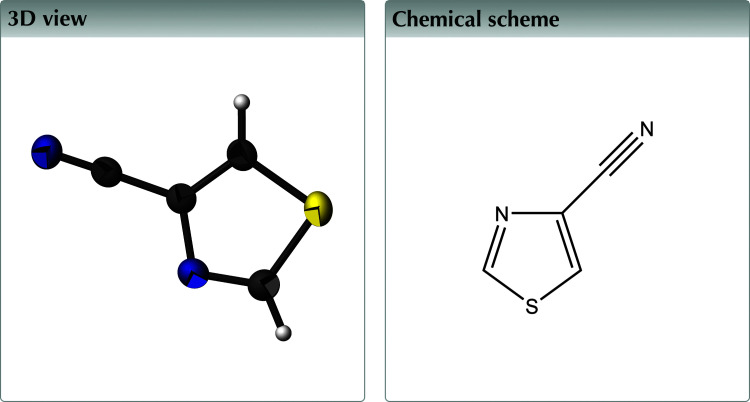

## Structure description

**Figure 1:**
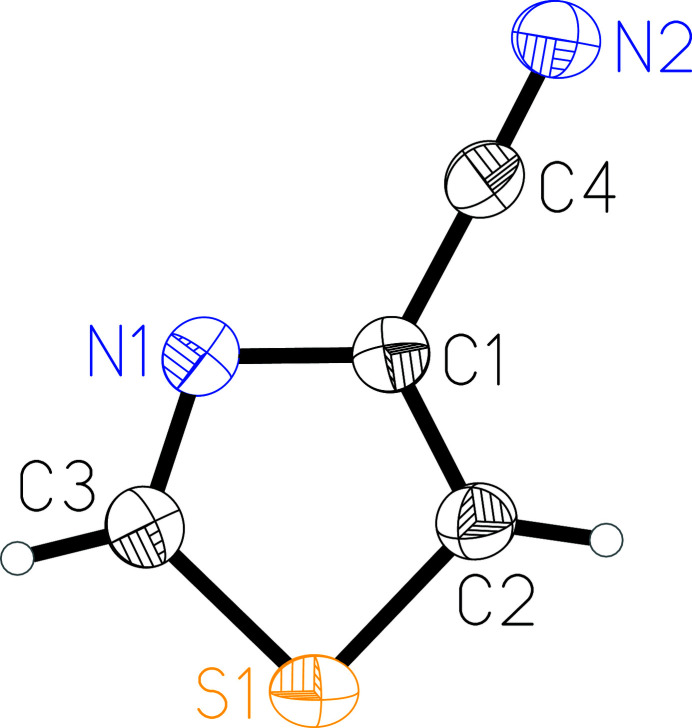
Mol­ecular structure of the title compound with atom labelling and displacement ellipsoids drawn at 50% probability level.

The title compound, C_4_H_2_N_2_S, consists of a 1,3-thia­zole ring substituted in the 4-position by a nitrile group (Fig. 1 ▸). The whole mol­ecule is nearly planar with a mean deviation from the best plane defined by all non-hydrogen atoms of 0.005 Å. All bond lengths are in the expected ranges (Allen *et al.*, 1987 ▸).

**Figure 2:**
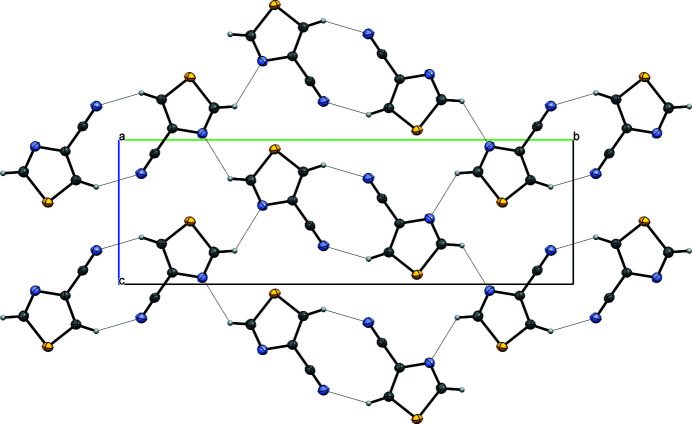
Packing diagram for the title compound along the *a* axis. Ellipsoids are drawn at the 30% probability level. Hydrogen bonds are shown as dotted lines.

In the crystal, weak C—H⋯N hydrogen bonds arising from both C—H groupings build up a wavy layer of mol­ecules in the (011) plane (Table 1 ▸, Fig. 2 ▸). The layers are stacked in the (100) direction by weak π–π stacking inter­actions between the 1,3-thia­zole rings [centroid–centroid distance = 3.7924 (10) Å, ring slippage = 1.39 Å].

## Synthesis and crystallization

Commercial powder of the title compound (Fluoro­chem, UK, catalogue No. # 076318) was purified by sublimation at normal pressure on a hot plate set to 55°C. The colourless crystals formed over two days on the covering watch glass. ^1^H NMR (300.2 MHz, DMSO-*d*_6_) *δ* 9.316, 9.310 (*J* = 1.82 Hz, H3), 8.908, 8.902 (*J* = 1.84 Hz, H2). ^13^C NMR (75.5 MHz, DMSO-*d*_6_) *δ* 157.4, 133.6, 125.9, 114.5. The NMR data are consistent with those previously published by Augustine *et al.* (2009 ▸).

## Refinement

Crystal data, data collection and structure refinement details are summarized in Table 2 ▸.

## Supplementary Material

Crystal structure: contains datablock(s) I. DOI: 10.1107/S2414314621013328/hb4395sup1.cif

Structure factors: contains datablock(s) I. DOI: 10.1107/S2414314621013328/hb4395Isup2.hkl

Supporting information file. DOI: 10.1107/S2414314621013328/hb4395Isup3.cml

CCDC reference: 2128844

Additional supporting information:  crystallographic information; 3D view; checkCIF report

## Figures and Tables

**Table 1:** Hydrogen-bond geometry (Å, °)

*D*—H⋯*A*	*D*—H	H⋯*A*	*D*⋯*A*	*D*—H⋯*A*
C2—H2⋯N2^i^	0.95	2.59	3.374 (2)	140
C3—H3⋯N1^ii^	0.95	2.57	3.257 (2)	129

Symmetry codes: (i) 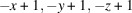 ; (ii) 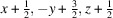 .

**Table 2:** Experimental details

Crystal data	
Chemical formula	C_4_H_2_N_2_S
*M*_r_	110.14
Crystal system, space group	Monoclinic, *P*2_1_/*n*
Temperature (K)	150
*a*, *b*, *c* (Å)	3.7924 (3), 19.8932 (18), 6.3155 (5)
β (°)	91.084 (6)
*V* (Å^3^)	476.37 (7)
*Z*	4
Radiation type	Cu *K*α
μ (mm^−1^)	4.77
Crystal size (mm)	0.24 × 0.18 × 0.08

Data collection	
Diffractometer	Bruker APEXII CCD
Absorption correction	Multi-scan (*SADABS*; Bruker, 2014 ▸)
*T*_min_, *T*_max_	0.40, 0.71
No. of measured, independent and observed [*I* > 2σ(*I*)] reflections	4709, 854, 783
*R*_int_	0.040
(sin θ/λ)_max_ (Å^−1^)	0.596

Refinement	
*R*[*F* ^2^ > 2σ(*F* ^2^)], *wR*(*F* ^2^), *S*	0.038, 0.097, 1.09
No. of reflections	854
No. of parameters	64
H-atom treatment	H-atom parameters constrained
Δρ_max_, Δρ_min_ (e Å^−3^)	0.32, −0.23

Computer programs: *APEX2* (Bruker, 2014 ▸), *SAINT* (Bruker, 2013 ▸), *SHELXS97* (Sheldrick, 2008 ▸), *SHELXL2018/3* (Sheldrick, 2015 ▸), *XP* in *SHELXTL* (Sheldrick, 2015 ▸), *Mercury* (Macrae *et al.*, 2020 ▸) and *publCIF* (Westrip, 2010 ▸).

## full crystallographic data

### Crystal data

**Table:**

C_4_H_2_N_2_S	*F*(000) = 224
*M**_r_* = 110.14	*D*_x_ = 1.536 Mg m^−^^3^
Monoclinic, *P*2_1_/*n*	Cu *K*α radiation, λ = 1.54178 Å
*a* = 3.7924 (3) Å	Cell parameters from 2588 reflections
*b* = 19.8932 (18) Å	θ = 4.5–66.7°
*c* = 6.3155 (5) Å	µ = 4.77 mm^−^^1^
β = 91.084 (6)°	*T* = 150 K
*V* = 476.37 (7) Å^3^	Plate, colourless
*Z* = 4	0.24 × 0.18 × 0.08 mm

### Data collection

**Table:**

Bruker APEXII CCD diffractometer	854 independent reflections
Radiation source: microfocus	783 reflections with *I* > 2σ(*I*)
Multilayer monochromator	*R*_int_ = 0.040
Detector resolution: 8.3333 pixels mm^-1^	θ_max_ = 66.7°, θ_min_ = 4.5°
φ and ω scans	*h* = −4→4
Absorption correction: multi-scan (SADABS; Bruker, 2014)	*k* = −23→23
*T*_min_ = 0.40, *T*_max_ = 0.71	*l* = −7→7
4709 measured reflections	

### Refinement

**Table:**

Refinement on *F*^2^	Primary atom site location: structure-invariant direct methods
Least-squares matrix: full	Hydrogen site location: inferred from neighbouring sites
*R*[*F*^2^ > 2σ(*F*^2^)] = 0.038	H-atom parameters constrained
*wR*(*F*^2^) = 0.097	*w* = 1/[σ^2^(*F*_o_^2^) + (0.0638*P*)^2^ + 0.0618*P*] where *P* = (*F*_o_^2^ + 2*F*_c_^2^)/3
*S* = 1.09	(Δ/σ)_max_ < 0.001
854 reflections	Δρ_max_ = 0.32 e Å^−^^3^
64 parameters	Δρ_min_ = −0.23 e Å^−^^3^
0 restraints	

### Special details

**Table:**

Geometry. All esds (except the esd in the dihedral angle between two l.s. planes) are estimated using the full covariance matrix. The cell esds are taken into account individually in the estimation of esds in distances, angles and torsion angles; correlations between esds in cell parameters are only used when they are defined by crystal symmetry. An approximate (isotropic) treatment of cell esds is used for estimating esds involving l.s. planes.

### Fractional atomic coordinates and isotropic or equivalent isotropic displacement parameters (Å^2^)

**Table:**

	*x*	*y*	*z*	*U*_iso_*/*U*_eq_	
C1	0.8382 (4)	0.61784 (9)	0.5796 (3)	0.0306 (4)	
C2	0.8897 (5)	0.59416 (9)	0.7795 (3)	0.0360 (4)	
H2	0.838664	0.549800	0.825466	0.043*	
C3	1.0479 (5)	0.70963 (10)	0.7216 (3)	0.0388 (5)	
H3	1.124522	0.755015	0.731394	0.047*	
C4	0.6933 (5)	0.57919 (9)	0.4057 (3)	0.0350 (4)	
N1	0.9271 (4)	0.68396 (9)	0.5450 (3)	0.0400 (4)	
N2	0.5794 (5)	0.54939 (9)	0.2660 (3)	0.0450 (4)	
S1	1.06029 (11)	0.65664 (2)	0.93452 (7)	0.0367 (2)	

### Atomic displacement parameters (Å^2^)

**Table:**

	*U*^11^	*U*^22^	*U*^33^	*U*^12^	*U*^13^	*U*^23^
C1	0.0280 (7)	0.0334 (9)	0.0306 (9)	0.0004 (7)	0.0030 (6)	0.0000 (6)
C2	0.0424 (10)	0.0352 (9)	0.0304 (9)	0.0020 (7)	0.0011 (7)	0.0000 (7)
C3	0.0400 (9)	0.0378 (9)	0.0383 (11)	−0.0060 (7)	−0.0022 (8)	0.0015 (7)
C4	0.0381 (9)	0.0364 (9)	0.0305 (9)	−0.0020 (7)	0.0040 (7)	0.0031 (7)
N1	0.0480 (10)	0.0372 (10)	0.0346 (9)	−0.0069 (6)	−0.0029 (7)	0.0049 (6)
N2	0.0559 (10)	0.0455 (9)	0.0336 (9)	−0.0102 (8)	0.0000 (7)	−0.0022 (7)
S1	0.0380 (3)	0.0426 (3)	0.0294 (3)	0.00213 (16)	−0.0020 (2)	−0.00236 (15)

### Geometric parameters (Å, º)

**Table:**

C1—C2	1.358 (3)	C3—N1	1.302 (3)
C1—N1	1.376 (3)	C3—S1	1.7089 (19)
C1—C4	1.441 (3)	C3—H3	0.9500
C2—S1	1.7024 (19)	C4—N2	1.141 (3)
C2—H2	0.9500		
			
C2—C1—N1	116.58 (16)	N1—C3—S1	115.85 (15)
C2—C1—C4	124.71 (17)	N1—C3—H3	122.1
N1—C1—C4	118.70 (16)	S1—C3—H3	122.1
C1—C2—S1	109.13 (14)	N2—C4—C1	178.96 (19)
C1—C2—H2	125.4	C3—N1—C1	108.81 (16)
S1—C2—H2	125.4	C2—S1—C3	89.62 (9)
			
N1—C1—C2—S1	−0.4 (2)	C4—C1—N1—C3	179.24 (16)
C4—C1—C2—S1	−179.26 (14)	C1—C2—S1—C3	0.28 (15)
S1—C3—N1—C1	−0.1 (2)	N1—C3—S1—C2	−0.12 (16)
C2—C1—N1—C3	0.3 (2)		

### Hydrogen-bond geometry (Å, º)

**Table:**

*D*—H···*A*	*D*—H	H···*A*	*D*···*A*	*D*—H···*A*
C2—H2···N2^i^	0.95	2.59	3.374 (2)	140
C3—H3···N1^ii^	0.95	2.57	3.257 (2)	129

Symmetry codes: (i) −*x*+1, −*y*+1, −*z*+1; (ii) *x*+1/2, −*y*+3/2, *z*+1/2.
